# Supplementary material for: Efficient Implementation of a Robot-Assisted Radical Cystectomy Program in a Naïve Centre Experienced in Open Radical Cystectomy and Other Robot-Assisted Surgeries: A Comparative Analysis of Perioperative Outcomes and Complications
Source: Cancers (Basel). 2025 Jul 31;17(15):2532. doi: 10.3390/cancers17152532 (PMC12345945; doi:10.3390/cancers17152532)
Supplement: Supplementary file 1 [file cancers-17-02532-s001.zip › Suppl_Table_S1_CORRECTED_PROOFS.pdf]

Supplementary Table S1. Quality criteria for accurate and comprehensive reporting of surgical outcomes recommended by the European Association of Urology Guidelines.

**Criteria**	
1. Define the method of accruing data	Prospective data collection based on patient charts or in-person or telephone interviews
2. Define who collected the data	Data were collected by medical staff
3. Indicate the duration of follow-up	Ninety days
4. Include outpatient information	Outpatient information was collected
5. Include mortality data and causes of death	Ninety-day mortality data and causes of death were collected
6. Include definitions of complications	Complications were defined as per the Clavien-Dindo classification
7. Define procedure-specific complications	Procedure-specific complications were defined
8. Report intraoperative and postoperative complications separately	Intraoperative and postoperative complications are reported separately
9. Use a severity grading system for postoperative complications	Complication severity was graded according to the Memorial Sloan-Kettering Cancer Center complication grading system
10. Postoperative complications should be presented in a table either by grade or by complication type	Complications are presented in a table by type and grade
11. Include risk factors	Risk factors for complications were investigated in an exploratory analysis
12. Include readmissions and causes	Readmissions and causes were reported
13. Include reoperations, types, and causes	Reoperations, types, and causes were reported
14. Include the percentage of patients lost to follow-up	No patients were lost to follow-up
