# Supplementary material for: Efficient Implementation of a Robot-Assisted Radical Cystectomy Program in a Naïve Centre Experienced in Open Radical Cystectomy and Other Robot-Assisted Surgeries: A Comparative Analysis of Perioperative Outcomes and Complications
Source: Cancers (Basel). 2025 Jul 31;17(15):2532. doi: 10.3390/cancers17152532 (PMC12345945; doi:10.3390/cancers17152532)
Supplement: Supplementary file 1 [file cancers-17-02532-s001.zip › Suppl_Table_S2_CORRECTED_PROOFS.pdf]

Supplementary Table S2. Summary of all postoperative complications classified by severity in the entire cohort of radical cystectomy patients.

**Complication**	**Overall (n=279)**	**Severe (n=51)**
Gastrointestinal	*50 (17.9%)*	*9 (17.6%)*
Clostridium Difficile colitis	1 (0.4%)	0 (0%)
Constipation	3 (1.1%)	0 (0%)
Diarrhoea	8 (2.9%)	0 (0%)
Nausea/Vomit	14 (5.0%)	0 (0%)
Ileus	15 (5.4%)	0 (0%)
Small bowel obstruction	6 (2.2%)	6 (11.7%)
Intestinal perforation	3 (1.1%)	3 (5.9%)
Infectious	*64 (22.9%)*	*9 (17.6%)*
Abdominal abscess	1 (0.4%)	1 (2%)
Fever of unknown origin	35 (12.5%)	0 (0%)
Pyelonephritis	3 (1.1%)	1 (2%)
Sepsis	13 (4.7%)	6 (11.7%)
Urinary tract infection	8 (2.9%)	0 (0%)
Endocarditis	1 (0.4%)	1 (2%)
SARS-CoV-2 test positivity	3 (1.1%)	0 (0%)
Wound	*13 (4.7%)*	*3 (5.9%)*
Wound dehiscence	3 (1.1%)	3 (5.9%)
Wound seroma	2 (0.7%)	0 (0%)
High output from pelvic drain	8 (2.9%)	0 (0%)
Genitourinary	*32 (11.5%)*	*19 (37.2%)*
Acute kidney injury	11 (3.9%)	11 (21.6%)
Gross upper tract haematuria	4 (1.4%)	1 (2%)
Ureteric obstruction	2 (0.7%)	2 (3.9%)

Urinary fistula	3 (1.1%)	0 (0%)
Ureteric catheter obstruction	6 (2.2%)	3 (5.9%)
Ureteric catheter dislocation	4 (1.4%)	2 (3.9%)
Nephrostomy tube dislocation	2 (0.7%)	0 (0%)
Cardiac	*24 (8.6%)*	*4 (7.8%)*
Arrhythmia	6 (2.2%)	0 (0%)
Pre-arrest	1 (0.4%)	1 (2%)
Pericarditis	1 (0.4%)	0 (0%)
Hypertensive crisis	9 (3.2%)	0 (0%)
Hypotensive crisis	4 (1.4%)	0 (0%)
Myocardial infarction	2 (0.7%)	2 (3.9%)
Acute heart failure	1 (0.4%)	1 (2%)
Pulmonary	*7 (2.5%)*	*2 (4%)*
Pleural effusion	3 (1.1%)	0 (0%)
Dyspnoea	2 (0.7%)	0 (0%)
Acute respiratory distress syndrome	2 (0.7%)	2 (3.9%)
Bleeding	*40 (14.3%)*	*1 (2%)*
Anaemia	38 (13.6%)	0 (0%)
Gastrointestinal bleeding	1 (0.4%)	0 (0%)
Pelvic bleeding	1 (0.4%)	1 (2%)
Thromboembolic	*6 (2.2%)*	*2 (3.9%)*
Deep vein thrombosis	1 (0.4%)	0 (0%)
Superficial vein thrombosis	1 (0.4%)	0 (0%)
Hypogastric artery thrombosis	1 (0.4%)	1 (2%)
Pulmonary embolism	3 (1.1%)	1 (2%)
Neurological	*11 (3.9%)*	*1 (2%)*
Epileptic seizure	1 (0.4%)	1 (2%)
Agitation/Delirium	6 (2.2%)	0 (0%)

Peripheral neuropathy	3 (1.1%)	0 (0%)
Hypomyotrophy	1 (0.4%)	0 (0%)
Blood test alterations	*27 (9.7%)*	*0 (0%)*
Hyperkalaemia	3 (1.1%)	0 (0%)
Hypokalaemia	9 (3.2%)	0 (0%)
Elevated renal function tests	6 (2.2%)	0 (0%)
Elevated liver function tests	1 (0.4%)	0 (0%)
Hyperglycaemia	3 (1.1%)	0 (0%)
Hypoglycaemia	1 (0.4%)	0 (0%)
Hypoalbuminemia	3 (1.1%)	0 (0%)
Hypoproteinaemia	1 (0.4%)	0 (0%)
Miscellaneous	*5 (1.8%)*	*1 (2%)*
Hypothermia	1 (0.4%)	0 (0%)
Hypercapnia	1 (0.4%)	0 (0%)
Bedsore	1 (0.4%)	0 (0%)
Fall	1 (0.4%)	0 (0%)
Femur fracture	1 (0.4%)	1 (2%)
